# Supplementary material for: The Chlamydia trachomatis-secreted effector protein CT181 binds to Mcl-1 and prolongs neutrophil survival
Source: mBio. 2026 May 15;17(6):e00357-26. doi: 10.1128/mbio.00357-26 (PMC13251465; doi:10.1128/mbio.00357-26)
Supplement: Supplemental figures — Fig. S1 to S5. [file mbio.00357-26-s0001.docx]

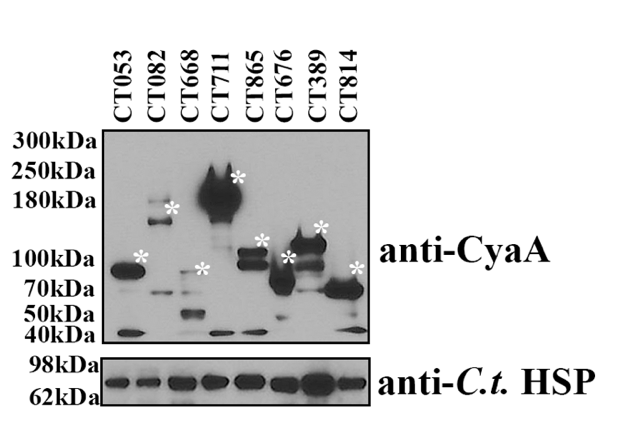


**Figure S1: Expression of candidate effector-CyaA fusion proteins in *C.t.*** Candidate secretion substrates were expressed as C-terminal fusions to the CyaA-tag and transformed into *C.t*. HeLa cells were infected at an MOI of 5 for 24h with each candidate, after which expression of the fusion protein was confirmed by immunoblotting with anti-CyaA antibodies. *C.t.* HSP-60 was used as a loading control. Asterisks on the right of each band indicate the product corresponding to the anticipated molecular weight of each CyaA fusion protein. Data are representative of three independent experiments.

**
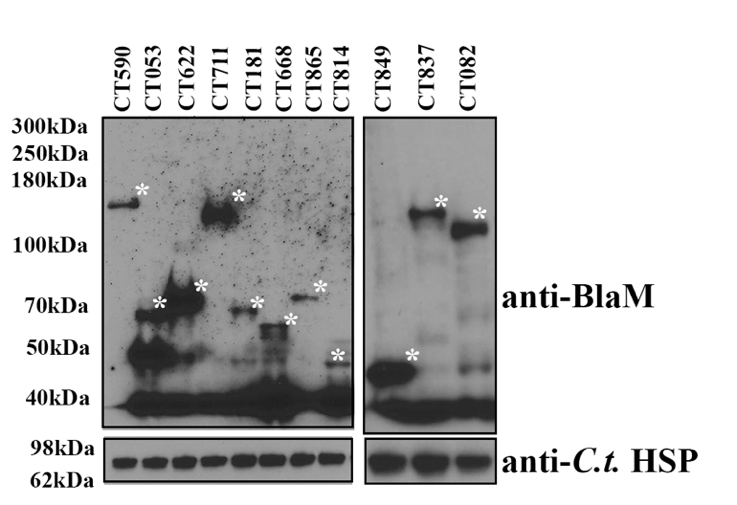
**

**Figure S2: Expression of candidate effector-BlaM fusion proteins in *C.t.*** Candidate secretion substrates were expressed as C-terminal fusions to the BlaM-tag and transformed into *C.t*. HeLa cells were infected at an MOI of 5 for 24h with each candidate, after which expression of the fusion protein was confirmed by immunoblotting with anti-BlaM antibodies. *C.t.* HSP-60 was used as a loading control. Asterisks on the right of each band indicate the product corresponding to the anticipated molecular weight of each BlaM fusion protein. Data are representative of three independent experiments.


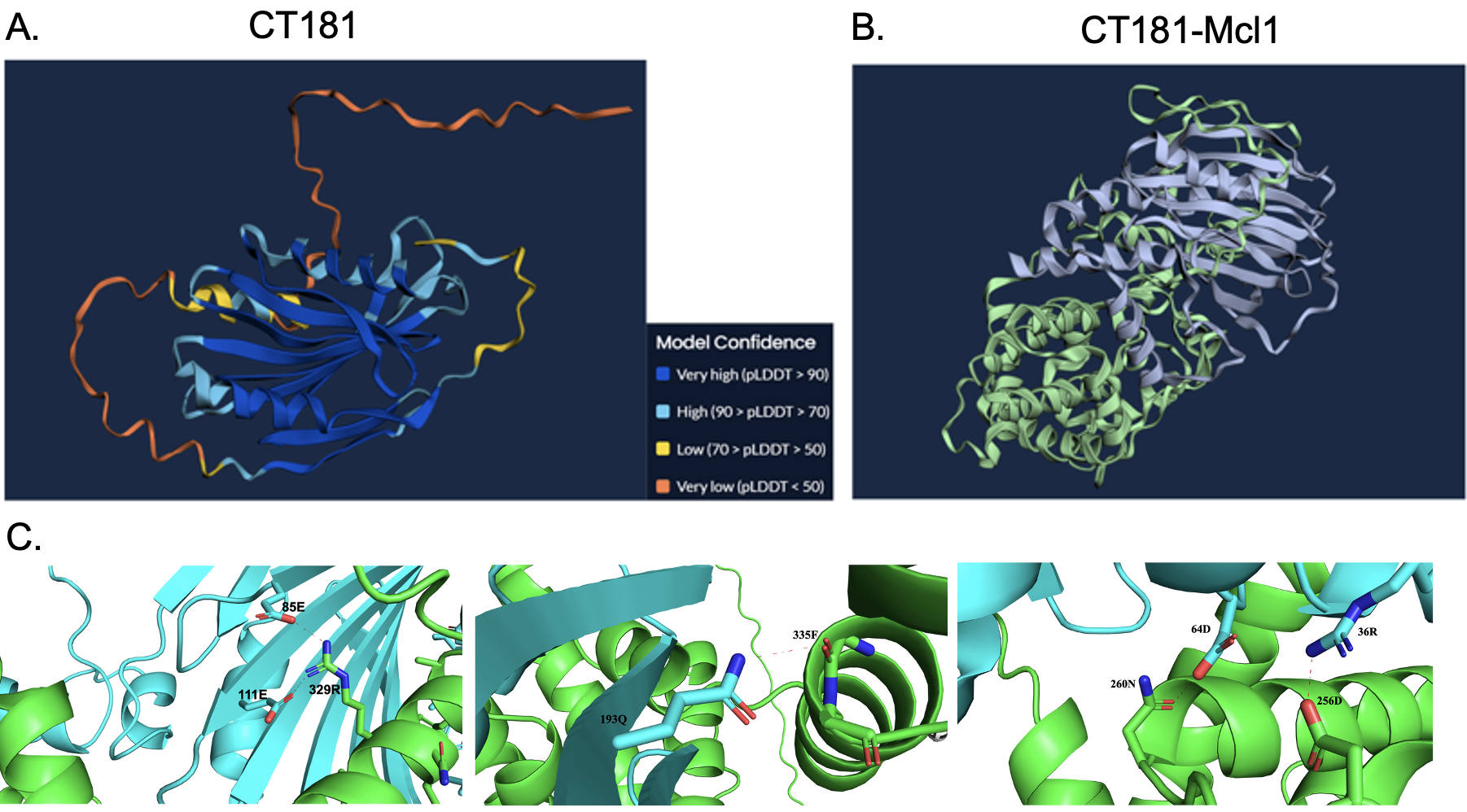


**Figure S3: Alphafold model of CT181 with and without Mcl-1*.*** (A) Alphafold predictions of CT181 alone or (B)with Mcl-1. CT181 is shown in purple and Mcl-1 is shown in green. (C) Protein protein interaction analysis of the modelled complex between CT181 and MCL1 revealed potential intermolecular contacts between residues 85E and 111E of chain B of CT181 (cyan) and 329R of chain A of MCL1 (green) (C-left). A second interaction was identified between the side chain of residue 193Q of CT181 and the peptide backbone of residue 335F of MCL1 (C-middle). Additional contacts were detected involving 64D and 36R of CT181 with 260N and 256D of MCL1 (D-right).

**A. B.**


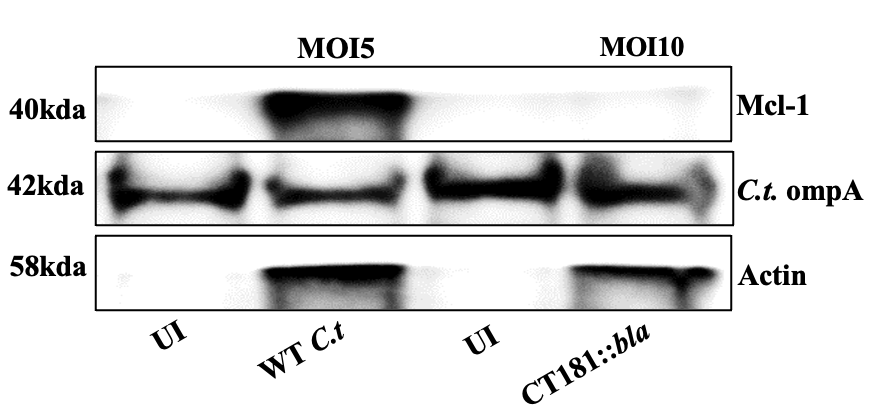


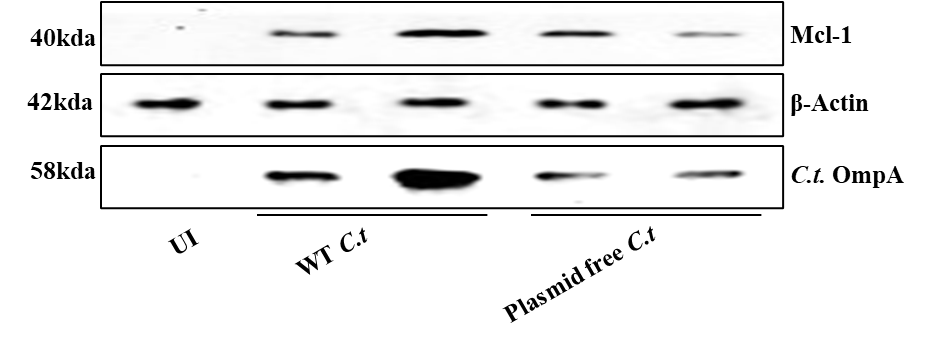


**C.**


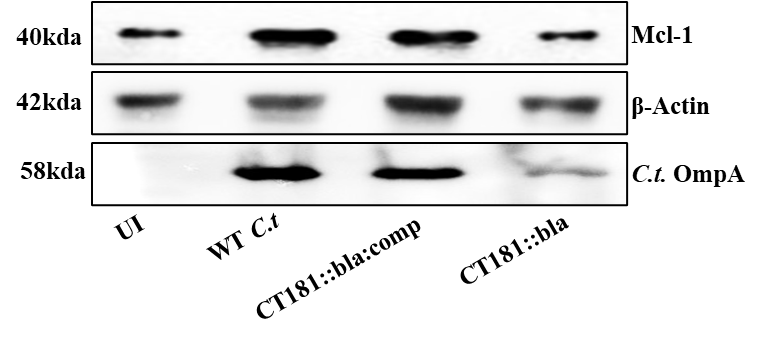


**Figure S4: CT181 mutant’s inability to stabilize Mcl-1 levels in human neutrophils is not dependent on the bacterial burden*.* (A-C**) Freshly isolated PMNs from human blood were either uninfected or infected at an MOI of 5. (**A**) To confirm Mcl-1 stabilization is not due to the lower burden associated with the CT181::*bla*, an MOI of 10 was used. (**B**) Mcl-1 and *C.t* OmpA expression were analyzed by western blot 48 hours post infection (h.p.i) with wild type (WT) *C.t* or Plasmid free *C.t*. (**C**) Mcl-1 and *C.t* OmpA expression were analyzed by western blot 48 h.p.i with either WT *C.t* or CT181::*bla* or CT181::*bla:comp*. The data are representative of three independent experiments.


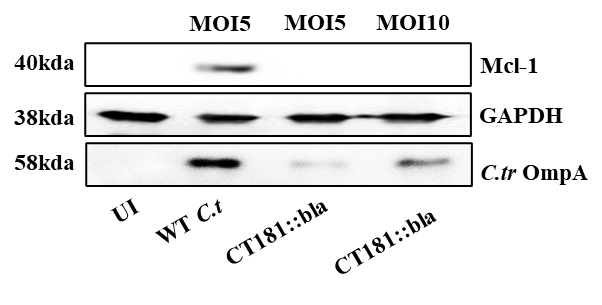
**A.**

**B.**

**
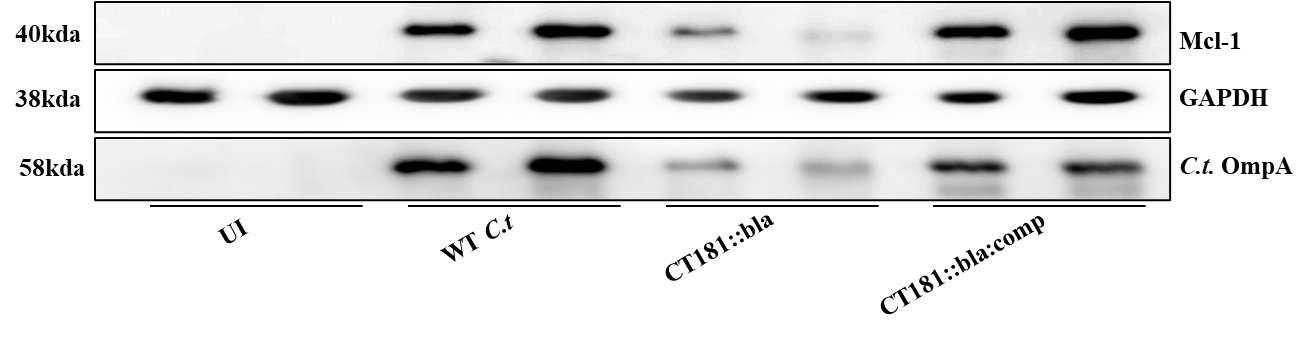
**

**Figure S5: CT181 mutant’s inability to stabilize Mcl-1 levels in mouse neutrophils is not dependent on the bacterial burden*.* (A-C**) Freshly isolated PMNs from human blood were either uninfected or infected at an MOI of 5. (**A**) To confirm Mcl-1 stabilization is not due to the lower burden associated with the CT181::*bla*, an MOI of 5 and 10 were used. (**B**) Mcl-1 and *C.t* OmpA expression were analyzed by western blot 48 h.p.i with either WT *C.t* or CT181::*bla* or CT181::*bla:comp*. Cropped image is provided in the Fig, 5B. The data are representative of three independent experiments.
