# Supplementary material for: The Chlamydia trachomatis-secreted effector protein CT181 binds to Mcl-1 and prolongs neutrophil survival
Source: mBio. 2026 May 15;17(6):e00357-26. doi: 10.1128/mbio.00357-26 (PMC13251465; doi:10.1128/mbio.00357-26)
Supplement: Table S1 — Primers. [file mbio.00357-26-s0002.docx]

Table S1: Primers used for completion of this study. Nucleotides in bold correspond to the restriction site. Nucleotides in blue correspond to the FLAG-tag.

| **Primer Name** | **Sequence** | **Uses** |
| --- | --- | --- |
| **Expression in *C.t.*** | | |
| CT053 NotI F | CC**GCGGCCGC**ATGAAAAGTGAGCGTTTAAAAAAATT | Secretion Assays |
| CT053 KpnI R | CC**GGTACC**CCATTCATTCGCGTCAGG | Secretion Assays |
| CT181 NotI F | CC**GCGGCCGC**ATGCTTTCAAAGTTCTGCAAACT | Secretion Assays |
| CT181 KpnI R | CC**GGTACC**TAAAGATACACGCTTTCTTATGT | Secretion Assays |
| CT365 NotI F | CC**GCGGCCGC**ATGGTTTCTAGGGTTCCTGGA | Secretion Assays |
| CT365 KpnI R | CC**GGTACC**CGATTCTACAAAAGAATCCCCAT | Secretion Assays |
| CT389 NotI F | CC**GCGGCCGC**ATGATGAAACCTCTACGTTTCGG | Secretion Assays |
| CT389 KpnI R | CC**GGTACC**GAAGCCATAACTTAATCGAAA | Secretion Assays |
| CT590 NotI F | CC**GCGGCCGC**ATGTCTCGTTTGGATGTTTCTGTA | Secretion Assays |
| CT590 KpnI R | CC**GGTACC**AAAAAAGCCTTTCGGCAAT | Secretion Assays |
| CT668 NotI F | CC**GCGGCCGC**ATGATAGATCCTCTTAAGCTTTT | Secretion Assays |
| CT668 KpnI R | CC**GGTACC**ACCTAACTGGGTTGTCATAATCG | Secretion Assays |
| CT676 NotI F | CC**GCGGCCGC**ATGGATCATACAGAAGAGTCTCCT | Secretion Assays |
| CT676 KpnI R | CC**GGTACC**GGGAGCATCGGTAGTATTC | Secretion Assays |
| CT814 NotI F | CC**GCGGCCGC**ATGTTCAGAAGCCAAAAA | Secretion Assays |
| CT814 KpnI R | CC**GGTACC**ATCTCGACGACTTTCATCAATAA | Secretion Assays |
| CT837 NotI F | CC**GCGGCCGC**ATGGAAAAGACTCGTAAG | Secretion Assays |
| CT837 KpnI R | CCG**GTACCA**ATACCTGAGAATTGCCACCC | Secretion Assays |
| CT865 NotI F | CC**GCGGCCGC**ATGATCAAGATAGCACAAAGTT | Secretion Assays |
| CT865 KpnI R | CC**GGTACC**TTTACAAAAACGGTCCCATAGATA | Secretion Assays |
| CT181 Flag KpnI R New | CC**GGTACCttacttatcgtcgtcatccttgtaatc**TAAAGATACACGCTTTCTTATGT | IP |
| CT181 KpnI F | CC**GGTACC**ATGCTTTCAAAGTTCTGCAAACT | Ectopic Expression |
| CT181 XhoI R | CC**CTCGAG**TAAAGATACACGCTTTCTTATGT | Ectopic Expression |
| CT181- 140/141s | TTCCCCTCTAGAAAAAAGCTTATAATTATCCTTAGGACACGACTATGTGCGCCCAGATAGGGTGTTAAGTCAAGTAGTTTAAGGTACTACTCTGTAAGATAACACAGAAAACAGCCAACCTAACCGAAAAGCGAAAGCTGATACGGGAACAGAGCACGGTTGGAAAGCGATGAGTTACCTAAAGACAATCGGGTACGACTGAGTCGCAATGTTAATCAGATATAAGGTATAAGTTGTGTTTACTGAACGCAAGTTTCTAATTTCGATTTGTCCTCGATAGAGGAAAGTGTCTGAAACCTCTAGTACAAAGAAAGGTAAGTTAGAATAGTCGACTTATCTGTTATCACCACATTTGTACAATCTG | gblock |
| Ct DNA F | GAAAGCAAGGGGAGCAAACAG | qPCR |
| Ct DNA R | GTACTCCTCAGGCGGCATAC | qPCR |
| Mouse GAPDH F | CCGTTGAATTTGCCGTGAGT | qPCR |
| Mouse GAPDH R | AGTGGCAAAGTGGAGATTGTTG | qPCR |
